# Public awareness, knowledge and practice relating to epilepsy amongst adult residents in rural Cameroon - case study of the Fundong health district

**DOI:** 10.11604/pamj.2013.14.32.2284

**Published:** 2013-01-22

**Authors:** Luchuo Engelbert Bain, Paschal Kum Awah, Innocent Takougang, Yelena Sigal, Tom T Ajime

**Affiliations:** 1 Centre For Population Studies and Health Promotion, CPSHP, BP 7535 Yaounde, Cameroon; 2 Hadassah Braun School of Public Health and Community Medicine, Hebrew University of Jerusalem, Israel; 3 Department of Anthropology, FALSS, University of Yaounde I, Cameroon; 4 Faculty of Medicine and Biomedical Sciences, University of Yaounde 1, Cameroon; 5 Faculty of Health Sciences, University of Buea, Cameroon

**Keywords:** Cameroon, rural, epilepsy, awareness, practice, Fundong

## Abstract

**Introduction:**

Epilepsy associated stigma remains a main hindrance to epilepsy care, especially in developing countries. In Africa, anti-epileptic drugs are available, affordable and effective. As of now, no community survey on epilepsy awareness and attitudes has been reported from this area Cameroon with a reported high prevalence of epilepsy.

**Methods:**

To contribute data to the elaboration of the National Epilepsy Control Programme, we carried out a cross-sectional descriptive community survey of 520 households. We had as main objective to obtain baseline data on the knowledge, attitudes and practice of adults towards epilepsy in rural Cameroon, and compare with existing data.

**Results:**

Most respondents had heard or read about epilepsy, knew someone who had epilepsy and had seen someone having a seizure. The most frequently cited cause of epilepsy was witchcraft. Most subjects believed epilepsy is contagious. Epilepsy was a form of madness or insanity to 33.5% of them. Only 54.9% of respondents would meet a medical doctor for the treatment. Most respondents would not permit equal employment opportunities, association and child's marriage to someone with epilepsy. Age, female sex and level of education were associated to negative attitudes (p<0.001).

**Conclusion:**

Adults in Fundong are very acquainted with epilepsy but have many erroneous beliefs about the condition. Their attitudes are generally negative. The National Epilepsy Programme must insist on modes of transmission, treatment options and first aid measures during epileptic seizures. The elderly (>50 years) and those without any formal education should be the main targets during health information, education and communication programmes.

## Introduction

Epilepsy is the most common serious neurological disorder. It is also one of the world's most prevalent non-communicable diseases [1, 2]. Studies in Africa have shown that the prevalence of epilepsy in the continent ranges between 15 per thousand and 25 per thousand [3]. One of the highest prevalence of epilepsy in the world, of over 60 per thousand has been recorded in the Mbam valley of the Centre province of Cameroon [4]. Misunderstanding about epilepsy, combined with the economic and financial barriers to availability of treatment in developing countries, play an important role in preventing treatment becoming available to millions of people in developing countries [5]. We therefore set out as the main objective of this study, to provide baseline data of the public knowledge, attitudes and practice towards epilepsy in the Fundong health district that could contribute to the ongoing efforts by the ministry of public health to set up a national program for the control of epilepsy.

## Methods

Fundong Health District is one of the largest of the 18 health districts of the North West Region of Cameroon. About 69Km from the Regional Headquarters Bamenda, has a population of about 300.000 inhabitants, mainly Christians and muslims on a total surface area of 145 Squared kilometers. Agricultural activities are the main source of income.

This study lasted for five (5) weeks, running from November 2008 to January 2009. We targeted a convenient sample of 520 households in the Fundong health area. Eight of the 16 villages were selected at random. Households in each of the areas were numbered and 65 households were finally selected from each of the areas by balloting for the interview. A 12 item pretested questionnaire was used for the interview. This questionnaire was adapted from other questionnaires for epilepsy surveys that had been done elsewhere: Tanzania, Northern Nigeria, Turkey, India, Vietnam and Cameroon (Batibo and Ebolowa). The questionnaire was divided into two portions; the first carrying sociodemographic data of the respondents including the age, sex, marital status, level of education, occupation and religion. The second part carried questions to evaluate familiarity, knowledge, attitudes and practice towards epilepsy. These questions were to know whether the patient had read or heard about the disease, or knew someone who had the disease, the cause of epilepsy, whether epilepsy was contagious. For their attitudes and understanding of the condition, the questionnaire evaluated whether respondents could allow their children to marry or associate with patients with epilepsy, whether epilepsy hindered patients from being employed in jobs normally like others, whether epilepsy was considered as a form of madness or insanity and the preferred treatment options. Most of the questions were simple open-ended questions requiring ‘yes’ or ‘no’ responses. Adults older than 18, who had been living in the district for over a year and were not known epileptics were included the study. Ethical clearance was obtained from the National Ethics Committees. A research authorization was gotten from the Fundong Divisional Office and the respective quarter heads in the selected villages. Informed consent was obtained from the respondents prior to the commencement of the interviews.

Data were entered using Epi Data Version 3.1 (www.epidata.dk, Odense, Denmark) and analyzed using the Epi Info Version 3.3.2 of the Centre for Disease Control, 2006. Chi square tests were used to measure association between sociodemographic data and each of the responses in univariate analysis. Frequencies were compared using Fischer's exact test. P - Values less than 0.05 were considered to be statistically significant.

## Results

**Figure 1:**
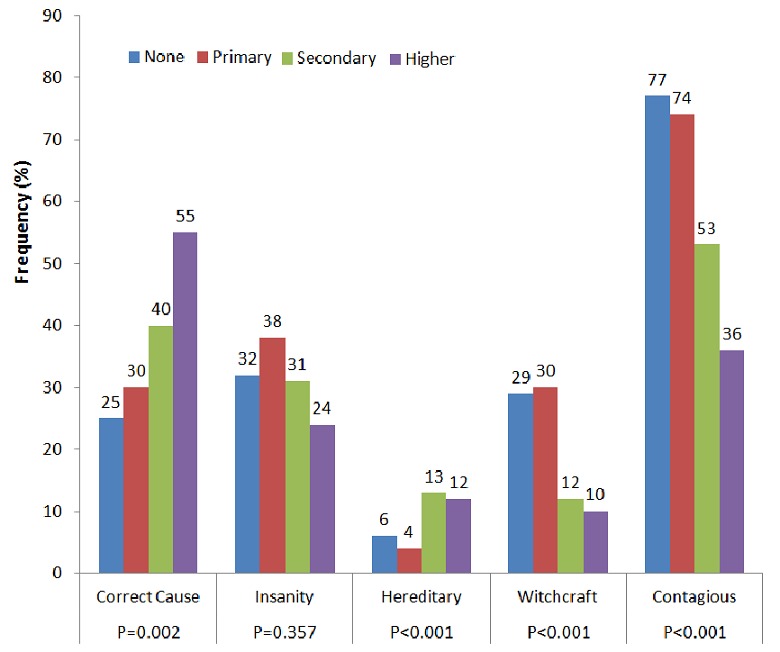
Level of education and understanding of epilepsy

Out of the 520 people contacted for the interview, 505 accepted to participate in the study giving a response rate of 97.1%. There were 273 (54, 1%) males and 232 (45.9%) females. In our sample population, 99.6% of respondents had heard or read about epilepsy, 86.1% knew someone who has or had epilepsy and 82.0% had ever seen someone having a seizure. Age greater 50 (p=0.021), female sex (p=0.040), having more than 5 children (p=0.046), higher level of education (p=0.039) and Christians (p=0.011) were more likely to know someone who has or had epilepsy. Illiteracy or low level of education, being a farmer and having more than 5 off springs were also associated to advocating witchcraft as cause of epilepsy (p<0.02) (Figure 1).

Christians (22.7%) had a greater tendency to associate epilepsy to witchcraft with respect to Muslims (13%), but the difference was not statistically significant. More than half (65.1%) of the respondents believed that epilepsy was a contagious disease. We also sought to know how it was transmitted for those who thought epilepsy was contagious. Over 38.6% of them said it through waste gas, 33.7% thought it could be transmitted by saliva, and 12.5% said it is through contact with blood and 9.7% considered sexual intercourse as the route of transmission.

Those who believed that epilepsy was contagious had a greater tendency to have the negative attitudes of refusing their children from getting married to (67.1%) (Table 1), or associating with PWE (60.5%) (p=0.001 and p < 0.0001 respectively). Those who considered epilepsy a form of madness were strongly against allowing their children from marrying PWE (69.8%), associating with them (60.4%) and only 30.2% of them would accord equal employment opportunities to epileptics and non-epileptics. (P values 0.005, 0.001 and < 0.0001 respectively).

**Table 1:** Socio-demographic background of respondents and their attitudes towards epilepsy

	Would you object if your offspring wanted to marry someone who had seizures?		Would you object to your children associating with people who sometimes had seizures?		Do you think people with epilepsy should be employed in jobs like other people?	
Socio-demographic characteristics	Yes (%)	No (%)	Yes (%)	No (%)	Yes (%)	No (%)
**Age group**						
18 – 29	64,98	35,2	49,4	50,6	52,9	47,1
30 - 49	55,9	44,1	51,4	48,6	48	52
50+	64,8	35,2	52,1	47,9	32,4	67,6
**Sex**						
Male	56,8	43,2	45,4	54,6	47,6	52,4
Female	67,5	32,5	56,5	43,5	49,1	50,9
**Marital status**						
Married	64	36	52,1	47,9	42,1	57,9
Divorced	60	40	66,7	33,3	46,7	53,3
Single	58,6	41,4	47,5	52,5	56,1	43,9
Widowed	75	25	50	50	37,5	62,5
**Level of education**						
None	68,5	31,5	58,4	41,6	47,2	52,8
Primary	64,2	35,8	53,1	46,9	35,8	64,2
Secondary	59,3	40,7	47,4	52,6	57,7	42,3
Higher	47,6	52,4	37,2	62,8	60,5	39,5
**Occupation**						
Wage earner	64,2	35,8	47,4	52,6	50,5	49,5
Farmer	62	38	53,7	46,3	41,5	58,5
Unemployed	55,2	44,8	52,2	47,8	41,8	58,2
**Religion**						
Christian	61,1	38,9	49,7	50,3	49,1	50,9
Muslim	78,3	21,7	69,6	30,4	30,4	69,6

Advanced age (p=0.017), being a Muslim (p=0.009), farming as occupation (p < 0.001) and a low level of education (p < 0.001) were associated with the belief that epilepsy is a contagious disease. Over 54.9% would recommend consultation with a medical doctor. However, 25.3% would opt for traditional medicine. Over 61.7% of our respondents would not allow their children to get married to someone who had epilepsy, 50.5% would object their children from associating with someone who sometimes had seizures and only 48.3% would offer equal. An increase in the level of education and being a student were associated with less discrimination in according jobs (p< 0.0001 and p=0.001 respectively). Knowing a correct cause of epilepsy was associated with more positive attitudes towards this condition. About 56.9% of them would offer equal job opportunities (p=0.002).

## Discussion

Familiarity with epilepsy in Fundong is high, with almost all adult having heard or read about the condition. Njamnshi et al have had similar findings in Batibo in the North West region of Cameroon [6]. Christians were more likely to know someone who had epilepsy (p=0.011), and to have witnessed an epileptic seizure (p < 0.0001) compared to Muslims. These findings tie with those of Kabir et al in Northern Nigeria, where a study in Kano, a typical Haussa state, only 26.5% of them had witnessed an epileptic seizure [7]. Our results cannot be conclusive since only 4.6% of the respondents were Muslims. Most of our subjects did not know what causes epilepsy (28.5%). Only 35.8% of the respondents knew a correct cause of epilepsy (i.e. Brain disease, birth defect or hereditary disease). This depicts a low knowledge of causes of epilepsy in our area of study. Knowing a correct of cause of epilepsy was associated with positive attitudes towards epileptics (P=0.002). Witchcraft was the most frequently cited cause of epilepsy (22.4%). False beliefs about the cause of epilepsy could be an indication of discrimination towards PWE in this area, and could also hinder access to health care. This false belief about epilepsy being contagious was more common among Muslims than Christians (p=0.009). Advanced age, low level of education and farmers had a greater tendency of holding this negative view (p=0.017, < 0.001 and < 0.001 respectively). The belief that epilepsy was a contagious disease was associated with discrimination towards People With Epilepsy, especially as far as marriage and associating with them was concerned (P<0.001 and p<0.0001 respectively). Given the high percentage (65.1 %) of the population who believed that epilepsy was contagious, this could reflect a serious problem of stigmatization towards PWE in this area.

In Hungaria, after a six year period of implementation of national guidelines for the acceptance and reintegration of PWE as part of the participation in the ‘Out of the shadows campaign’ of the International Bureau for Epilepsy, (IBE), International League for the Fight Against Epilepsy, ILAE and the World Health Organization, a significant reduction in negative attitudes were noted. There was a very significant increase in favouring equal employment opportunities (p^8^]. Caveness reported similar positive trends in the United States of America [9]. Continuous sensitization campaigns in Fundong on epilepsy could contribute to reduce the prevalence of these negative attitudes and misconceptions.

## Conclusion

This study reveals a high level of acquaintance with epilepsy amongst adults in Fundong. Negative attitudes towards people with this condition, and consequently discrimination against people with epilepsy remain a major concern. Any education programme should have those aged above 50, females and those who have not received any form of formal education as the main targets. A prevalence study is required in the Fundong Health District to ascertain the actual prevalence of epilepsy in this area. Regular sensitization campaigns on epilepsy are compelling in this area. The Socio cultural background and beliefs of each society determine attitudes towards epilepsy. Due to the rich cultural diversity of Cameroon, other studies should be carried out in many more areas of the country for a comprehensive and holistic approach in the implementation of control strategies of the National Epilepsy Control Program. Large scale studies in urban areas of the country should be envisaged.

### Limitations

Respondents could easily be giving socially acceptable responses, which might not reflect the real situation in the community. The quantitative nature of this study limits in depth exploration of the actual intensity of this condition, and interactions between the various socio-cultural determinants of epilepsy associated stigma. In depth interviews and focal group discussions in analyzing these issues, especially amongst epileptics, who were excluded from this study, could be more desirable.

## References

[1] Sander JW (2003). The epidemiology of epilepsy revisited. Current Opinion in Neurology..

[2] Saraceno B (2002). The WHO World Health Report 2001 on mental health. Epidemiology and Psychiatry Society..

[3] Sander JW (2003). The epidemiology of epilepsy revisited. Current Opinion in Neurology..

[4] Njamnshi AK, Dongmo L, Sini V (2002). Epilepsy in rural Cameroon: the alarming prevalence rates in the Mbam valley, presented during the 169th conference of the Swiss Society of Neurology in Zoug (CH).

[5] Burneo JG, Tellez-Zenteno J, Wiebe S (2005). Understanding the burden of epilepsy in Latin America: a systematic review of its prevalence and incidence. Epilepsy Research..

[6] Njamnshi AK, Angwafor SA, Tabah EN, Jallon P, Muna WF (2008). General public knowledge, attitudes, and practices with respect to epilepsy in the Batibo Health District, Cameroon.

[7] Kabir M, Iliyasu Z, Abubakar IS, Kabir ZS, Farinyaro AU (2005). Knowledge, attitude and beliefs about epilepsy among adults in a northern Nigerian urban community. Annals of African Medicine..

[8] Mirnics Z, Czikora G, Zavecz T, Halasz P (2000). Changes in Public attitudes toward epilepsy in Hungary: Results of surveys conducted in 1994 and 2000. Epilepsia.

[9] Caveness WF (1959). Trend in Public Attitudes toward Epilepsy over the Past Decade. Epilepsia.

